# Control of trichome branching by Chromatin Assembly Factor-1

**DOI:** 10.1186/1471-2229-8-54

**Published:** 2008-05-13

**Authors:** Vivien Exner, Wilhelm Gruissem, Lars Hennig

**Affiliations:** 1 Institute of Plant Sciences & Zurich-Basel Plant Science Center, ETH Zurich, CH-8092 Zurich, Switzerland

## Abstract

**Background:**

Chromatin dynamics and stability are both required to control normal development of multicellular organisms. Chromatin assembly factor CAF-1 is a histone chaperone that facilitates chromatin formation and the maintenance of specific chromatin states. In plants and animals CAF-1 is essential for normal development, but it is poorly understood which developmental pathways require CAF-1 function.

**Results:**

Mutations in all three CAF-1 subunits affect Arabidopsis trichome morphology and lack of CAF-1 function results in formation of trichomes with supernumerary branches. This phenotype can be partially alleviated by external sucrose. In contrast, other aspects of the CAF-1 mutant phenotype, such as defective meristem function and organ formation, are aggravated by external sucrose. Double mutant analyses revealed epistatic interactions between CAF-1 mutants and *stichel*, but non-epistatic interactions between CAF-1 mutants and *glabra3 *and *kaktus*. In addition, mutations in CAF-1 could partly suppress the strong overbranching and polyploidization phenotype of *kaktus *mutants.

**Conclusion:**

CAF-1 is required for cell differentiation and regulates trichome development together with STICHEL in an endoreduplication-independent pathway. This function of CAF-1 can be partially substituted by application of exogenous sucrose. Finally, CAF-1 is also needed for the high degree of endoreduplication in *kaktus *mutants and thus for the realization of *kaktus*' extreme overbranching phenotype.

## Background

Chromatin stability and dynamics have to be well balanced to guarantee normal development. While flexibility of the chromatin structure permits developmental transitions necessary during the life cycle of an organism, epigenetic as well as genetic information has to be reliably propagated within a certain developmental phase. Various protein complexes have been described to be involved in chromatin regulation [1-3]. One biochemically well characterized complex involved in chromatin replication is Chromatin Assembly Factor CAF-1, which deposits histones H3 and H4 in a replication-dependent manner onto DNA (for review see [4,5]. This complex was initially identified as a negative supercoiling-inducing factor in human cell extracts [6,7] and is conserved among all major eukaryotic lineages. Homologs have been found in yeast (subunits CAC1, CAC2, CAC3; [8], in mammals (p150, p60, p48; [9], in insects (p180, p105/75, p55; [10-12] and in plants (FASCIATA (FAS) 1, FAS2, MSI1; [13,14].

Yeast CAF-1 mutants have impaired maintenance of silencing at mating type loci and near the telomeres, and exhibit increased sensitivity towards ultraviolet radiation [8,15-20]. In higher eukaryotes, CAF-1 is specific for replication-coupled deposition of the H3.1 variant, while other histone chaperones deposit the H3.3 variant (called H3.2 in plants) in a replication-independent way [21,22]. Because mostly H3.3 and much less H3.1 is found in active chromatin [23], it has been proposed that CAF-1-mediated assembly of chromatin facilitates transcriptional repression through H3.1 deposition [24]. A recent report that H3.1-containing nucleosomes are more stable than H3.3-containing nucleosomes supports this model [25]. Replication-coupled deposition of H3.1 by CAF-1 is essential in metazoans, because loss of CAF-1 function causes severe defects in chromatin metabolism and eventual cell death in mouse and human cells [26-30]. Loss of CAF-1 causes developmental arrest in *Xenopus laevis *[31], Drosophila [32] and zebrafish [33].

*Arabidopsis thaliana *is the only higher eukaryote for which viable CAF-1 mutants are available (for review see [34]). Mutants deficient in FAS1 and FAS2, the two larger subunits of Arabidopsis CAF-1, were originally isolated for their altered phyllotaxis and their flattened and bifurcated stems [35,36], which is a phenotype known as fasciation [37]. Fasciation is associated with altered expression of *WUSCHEL*, which is a key regulatory gene that defines the stem cell niche in the shoot apical meristem (SAM) [13]. Misspecification of the *WUSCHEL *domain alters size and shape of the meristem, which subsequently changes primordia spacing and therefore causes distortion of phyllotaxis. In contrast to null mutants of *FAS1 *and *FAS2 *that are viable null mutants of the smallest CAF-1 subunit MSI1 are lethal [38]. This lethality is not caused by loss of CAF-1 function, however, but by loss of the FERTILIZATION INDEPENDENT SEED DEVELOPMENT (FIS) complex, of which MSI1 is a subunit as well [39].

Initial research with *fas *mutants focused on CAF-1 function in meristematic tissue [13,35,36] Recent studies showed, however, that CAF-1 is also needed for complete compaction of heterochromatin and maintenance of transcriptional gene silencing [40,41], homologous recombination [42,43], regulation of endoreduplication [34], and cell differentiation [44].

Trichomes or leaf hairs protrude from the leaf surface to protect the plant against adverse environmental conditions and herbivorous insects [45,46]. Depending on the plant species and function, trichomes are uni- or multicellular, metabolically active or inactive structures. In *Arabidopsis thaliana*, trichomes are single, living cells with a complex structure, which makes them well suited to study cell determination and differentiation. Trichomes originate from the epidermal cell layer and are evenly spaced by lateral inhibition (for an overview see: [47]). After determination, the trichome progenitor cell stops division and switches to endoreduplication. The cell enlarges and protrudes from the epidermal cell layer. On rosette leaves, two branching events give trichomes their characteristic three-ended morphology. Genetic analyses have revealed a complex regulatory network that controls trichome spacing and differentiation. Two major groups of genes control branching. Some of the genes influence branching directly, while others control branch number in an endoreduplication-dependent manner (reviewed by: [48]).

We have previously reported that trichome differentiation requires a functional CAF-1 complex, but it remained open in which genetic pathway CAF-1 acts during this process [44]. Here we provide evidence that CAF-1 and *STICHEL (STI)*, which encodes a protein with similarity to ATP-binding eubacterial DNA-polymerase III-subunits [49], together control trichome differentiation in an endoreduplication-independent pathway.

## Results

### Sucrose suppresses the CAF-1 mutant trichome phenotype

**Figure 1:**
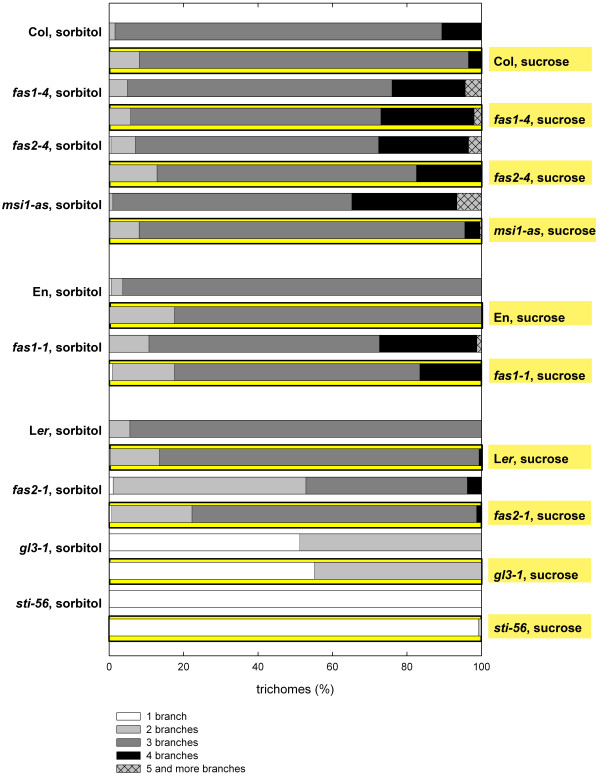
**Sucrose influences trichome morphology in CAF-1 mutants**. The overbranching of rosette leaf trichomes in CAF-1 mutants is reduced on medium containing sucrose. Trichome branch number was assessed on the first and second primary leaves of wild-types Col (294, 342), En (161, 136) and L*er *(119, 449) and the mutants *fas1-4 *(223, 171), *fas2-4 *(164, 121), *fas1-1 *(123, 98), *fas2-1 *(66, 124),*gl3-1 *(42, 55) and *sti*-*56 *(129, 171). Figures in parentheses represent the number of trichomes analyzed on sorbitol and sucrose, respectively. Note that *gl3-1 *produces only a limited number of trichomes on the primary rosette leaves. Plants were grown on MS medium supplied with either 1% sorbitol (unmarked bars) or 1% sucrose (bars highlighted in yellow).

During the analysis of trichome development in CAF-1 mutants we observed that *fas2*-*1 *seedlings had fewer trichomes with supernumerary branches when grown on MS medium containing sucrose than on MS medium alone (data not shown). Carbohydrates control cell cycle activity and are known to influence plant development and organ formation (for review see: [50,51]), but a role in trichome development has not been reported. To test whether sucrose generally influences trichome development, wild type and CAF-1 mutant plants were grown on MS medium with 1% sucrose. Control plants were grown on MS medium containing 1% of the non-metabolizable sugar sorbitol. The number of trichome branches was recorded for the first and second rosette leaves (Fig. 1). In wild type plants of Columbia (Col), Enkheim (En) and Landsberg *erecta *(L*er*) accessions, sucrose caused a small but consistent shift towards trichomes with fewer branches. This decrease in branch number was statistically significant (chi-squared test, p < 0.05) for Col, *fas2-4*, *msi1-as*, En and *fas2-1*. In CAF-1 mutants, sucrose suppressed, at least partially, the supernumerary branch phenotype. The effect was strongest in *msi1-as*, and weakest in *fas1-4*. Mutations of *STI *and *GLABRA3 (GL3)*, which positively regulate trichome branching through the endoreduplication-independent and endoreduplication-dependent pathway, respectively, usually produce trichomes without branching (*sti*) or only a single branching event (*gl3*). Both mutants were unaffected by sucrose (Fig. 1). Thus, sucrose affects branching during trichome differentiation and can partially substitute for loss of CAF-1.

### Sucrose does not generally attenuate CAF-1 mutant phenotypes

**Figure 2:**
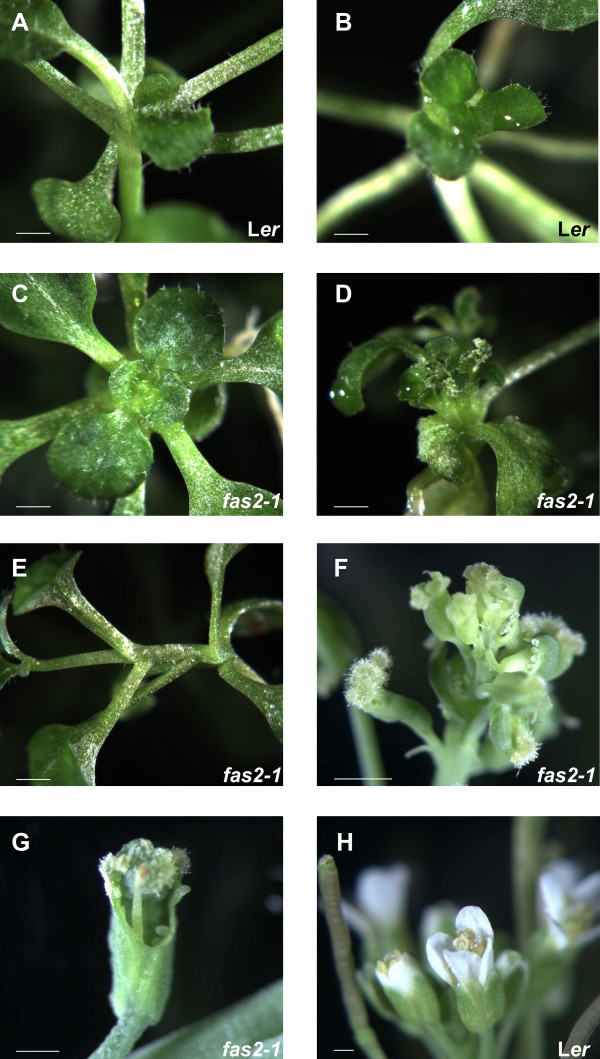
**Sucrose influences the phenotype of *fas2-1 *mutants**. Wild-type L*er *and *fas2*-1 mutant seedlings were grown on medium supplied with 1% sorbitol or with 1% sucrose. A: L*er *grown on sorbitol. B: L*er *grown on sucrose. C: *fas2-1 *grown on sorbitol. D: *fas2-1 *grown on sucrose. Note the severely distorted phyllotaxis. E: *fas2-1 *grown on sucrose exhibiting internode elongation. F and G: Flowers of *fas2-1 *plants grown for 2.5 weeks on sucrose and later on soil. H: Flowers of a L*er *plant grown for 2.5 weeks on sucrose and later on soil. Scale bars: 0.5 mm.

It is possible that sucrose generally suppresses CAF-1 mutant phenotypes. Detailed analysis of CAF1 mutants showed, however, that only trichome branching but not other aspects of the CAF-1 mutant phenotype were attenuated by sucrose. In fact, distortion of phyllotaxis was strongly enhanced in *fas2-1 *mutants grown on MS medium with sucrose (Fig. 2). The angles between successive leaves were highly irregular, and some primordia did not complete differentiation into leaves but showed weak radialization (data not shown). In addition, internodes elongated and the usual compact appearance of a rosette was lost (Table 1). Furthermore, even after *fas2-1 *seedlings were transferred from sucrose medium to soil, about 10% of the plants showed defects in flower development (Fig. 2F, G). These plants produced flowers with missing or severely malformed petals and stamens, and unfused carpels. Additionally, ectopic ovules were sometimes produced at the margin of cauline leaves. Such phenotypes were not observed in control plants. This strong enhancement of the mutant phenotype was not observed in the *fas1-1*, *fas1-4 *and *fas2-4 *CAF-1 mutant alleles, suggesting that L*er *is especially sensitive to loss of CAF-1 function when additional factors such as sucrose perturb early development.

**Table 1:** Sucrose strongly alters the phenotype of *fas2-1 *but not of the other CAF-1 mutants or wild-type plants.

Genotype, treatment	Wild-type phenotype		*fas *mutant phenotype, rosette habit		*fas *mutant phenotype, elongated internodes	
	
	Plants	%	Plants	%	Plants	%
En, sorbitol	14	93.3	1	6.7	0	0
En, sucrose	38	97.4	1	2.6	0	0
*fas1-1*, sorbitol	0	0	17	100	0	0
*fas1-1*, sucrose	0	0	36	100	0	0
L*er*, sorbitol	27	100	0	0	0	0
L*er*, sucrose	39	97.5	0	0	1	2.6
*fas2-1*, sorbitol	0	0	29	100	0	0
*fas2-1*, sucrose	0	0	26	18.3	116	81.7
Col, sorbitol	38	100	0	0	0	0
Col, sucrose	36	100	0	0	0	0
*fas1-4*, sorbitol	0	0	35	100	0	0
*fas1-4*, sucrose	0	0	37	100	0	0
*fas2-4*, sorbitol	0	0	25	100	0	0
*fas2-4*, sucrose	0	0	39	100	0	0
*msi1-as*, sorbitol	0	0	44	100	0	0
*msi1-as*, sucrose	0	0	42	100	0	0

Shown are the number of seedlings scored in a given category and the percentage.

### Mutations in CAF-1 partially suppress the *kaktus *supernumerary branching phenotype

**Figure 3:**
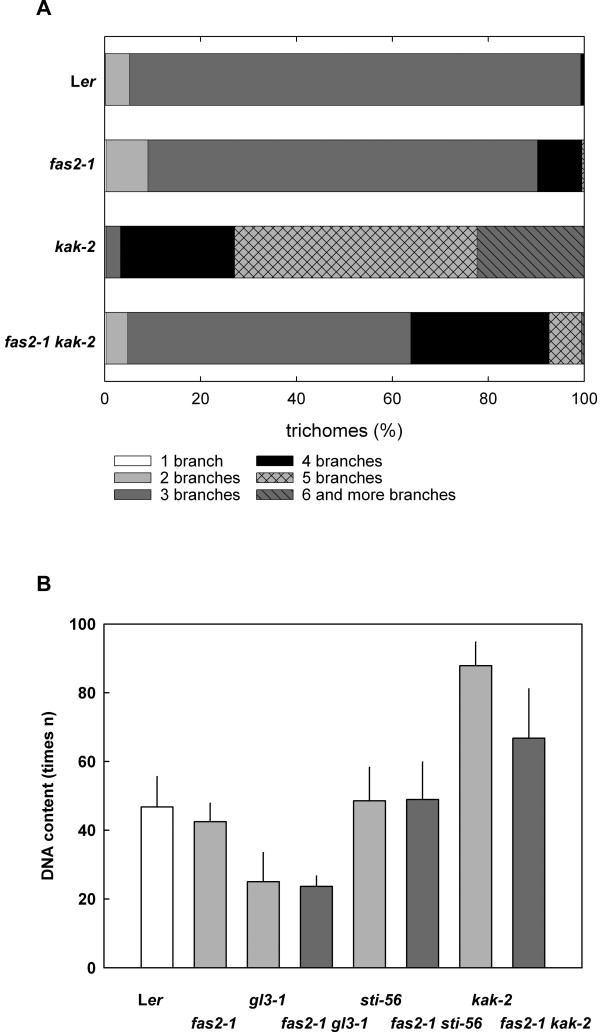
**Trichome phenotype in *fas2-1 kak-2 *double mutants**. A: Trichome branching in L*er*, *fas2*-1, *kak-2 *and *fas2-1 kak-2*. The double mutants have an intermediate number of branches per trichome compared to the single mutants. B: Nuclear DNA content of trichomes from L*er*, *fas2-1*, *kak-2 *and *fas2-1 kak-2 *leaves. The DNA content of trichome nuclei of *fas2-1 kak-2 *mutants is in between the DNA content of the single mutants.

We previously suggested that CAF-1 controls trichome branching via an endoreduplication-independent pathway [44]. To further test this hypothesis, we first analyzed *fas2-1 kak-2 *double mutants. *KAKTUS (KAK) *encodes a putative HECT-domain E3 ligase [52], and *kak *mutant trichomes have increased ploidy levels and highly supernumerary branches [53]. Characterization of the trichome morphology on rosette leaves of *fas2-1 kak-2 *double mutant plants revealed that the two alleles were not epistatic (Fig. 3A). This result is consistent with the hypothesis that CAF-1 controls trichome branching independent of the *KAK*-containing pathway. However, the branching phenotype of *fas2-1 kak-2 *trichomes was intermediate to the two single mutants rather than additive, suggesting that KAK and CAF-1 can influence each other.

### Loss of CAF1 function restricts DNA endoreduplication in *kak-2 *mutants

While *fas2-1 *mutants and wild-type plants have the same DNA content of trichome nuclei [44], mutations in *KAK *allow additional rounds of endoreduplication in leaf hair nuclei [53]. However, CAF-1 function is needed for chromatin integrity and has been suggested to be required during cell cycle progression [40]. It was therefore possible that loss of CAF-1 function in the *fas2-1 kak-2 *mutant restricts the *kak *endoreduplication potential and thus limits trichome branching in the *fas2-1 kak-2 *mutant. Analysis of the DNA content revealed that trichomes of *fas2-1 kak-2 *mutants had on average one third less nuclear DNA than trichomes of *kak-2 *single mutants (Fig. 3B). This level was between the numbers of endocycles observed in *fas2-1 *and *kak-2*. One possible explanation is that CAF-1 is needed for efficient progression through the endocycle in trichomes. Such a limitation would be consistent with the proposed slower progression through S-phase in CAF-1 mutants [40,54].

### CAF-1 and STICHEL act together in the endoreduplication-independent pathway of trichome differentiation

**Figure 4:**
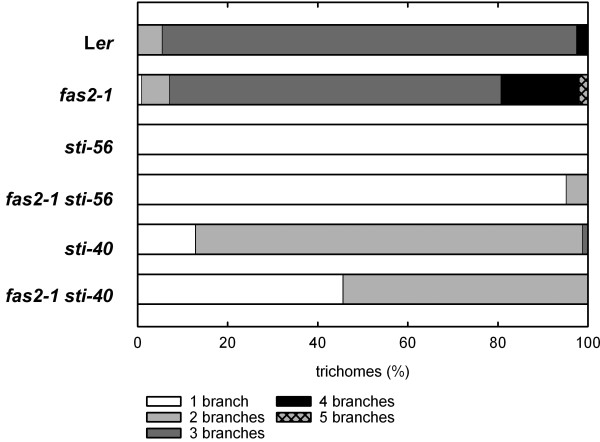
**Mutations in *STI *are epistatic over *fas2***. Trichome branching in L*er*, *fas2*-1, *sti-40*, *sti-56*, *fas2*-1 *sti-40 *and *fas2*-1 *sti-56*. Double mutants between two *sti *alleles and *fas2-1 *exhibit the same branching phenotype as the two *sti *alleles alone.

Analysis of *fas2-1 kak-2 *(this work) and *fas2-1 gl3-1 *[44] double mutants suggested that *FAS2 *acts in a pathway parallel to *KAK *and *GL3 *and controls trichome branching in an endoreduplication-independent manner. STICHEL (STI), a protein with similarity to eubacterial DNA-polymerase III-subunits [49], also controls trichome branching in an endoreduplication-independent pathway. To test whether CAF-1 functions in the STI-pathway for trichome differentiation, *fas2-1 *was crossed with a strong and a weak *sti *allele. While *sti-56 *almost completely abolishes trichome branching, *sti-40 *develops many trichomes with one branching event [49,55]. Analysis of trichome morphology of the *fas2 sti *double mutants revealed strong, although not complete epistasis of the *sti-56 *null allele over *fas2 *(Fig. 4). Interestingly, *fas2 *fortifies the weak phenotype of the hypomorphic *sti-40 *allele. Together, these results suggest that *FAS2 *and *STI *function together in the same pathway for trichome differentiation.

**Figure 5:**
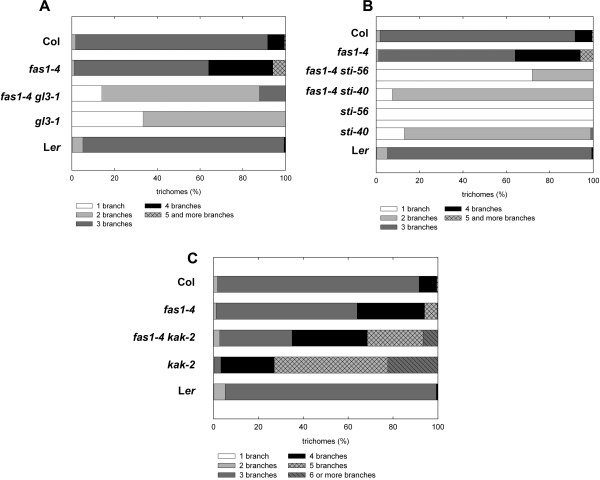
**Genetic interactions of *fas1-4 *with *gl3-1*, *si-40*, *sti-56 *and *kak-2***. A: Trichome branching on rosette leaves of *fas1-4*, *gl3-1 *and *fas1-4 gl3-1*. The double mutant is intermediate to the single mutants. B: Trichome branching on rosette leaves of *fas1-4*, *sti-40*, *sti-56*, *fas1*-4 *sti-40 *and *fas1-4 sti-56*. The two *sti *alleles are epistatic over *fas1-4*. C: Trichome branching on rosette leaves of *fas1-4*, *kak-2 *and *fas1-4 kak-2*. The strong overbranching phenotype of *kak-2 *is partially suppressed by *fas1-4*.

We have reported earlier that FAS2 controls trichome branching in the context of the CAF-1 complex [44]. To test the hypothesis that CAF-1 and *STI *function in the same pathway, we generated double mutants of *fas1 *and *gl3, kak *and *sti*. The trichome branching phenotypes of the various double mutants with *fas1 *and *fas2 *were similar: *fas1-4 gl3-1 *exhibited intermediate phenotypes (Fig. 5A) compared with the single mutants, while *fas1-4 sti-40 *and *fas1-4 sti-56 *again showed strong epistasis of *sti *over *fas1 *(Fig. 5B). Furthermore, *fas1-4 kak-2 *double mutants had a similar partial suppression of the *kak *phenotype as did the *fas2-1 kak-2 *double mutants (Fig. 5B). These results are consistent with our view that CAF-1 and *STI *function in the same pathway of trichome differentiation.

**Figure 6:**
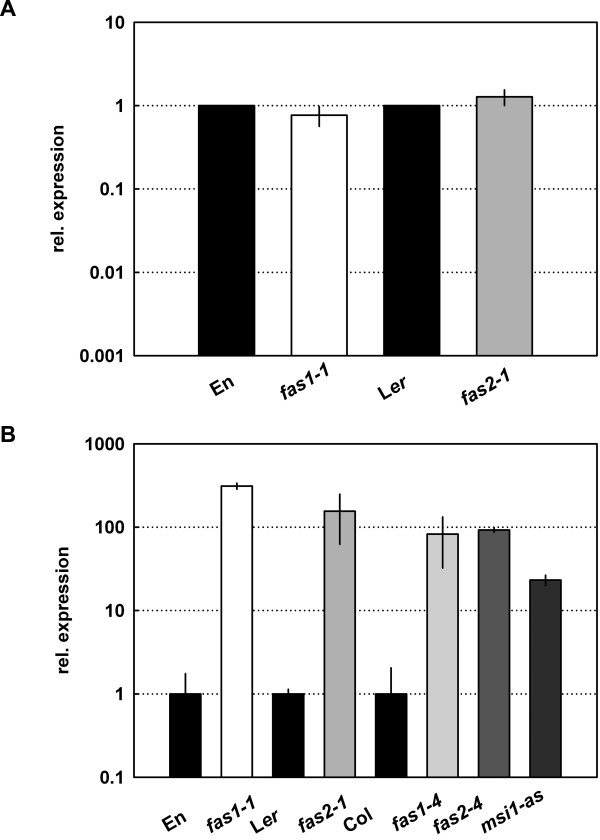
***H3.2 *but not *STI *expression is changed in *fas1-1 *and *fas2-1 *trichomes**. A: *STI *transcript levels were measured by quantitative RT-PCR in En, L*er*, *fas1-1 *and *fas2-1*. Expression is shown relative to the corresponding wild type. B: *H3.2 *transcript levels were measured by quantitative RT-PCR in En, L*er*, Col, *fas1 *and *fas2 *mutants and *msi1-as*. Expression is shown relative to the corresponding wild type.

Because *sti *showed epistasis over CAF-1 mutant alleles, it is likely that STI acts downstream of CAF-1. One possibility is that CAF-1 is needed for correct *STI *expression during trichome differentiation. To test this hypothesis we measured *STI *mRNA levels in CAF-1 mutants by quantitative RT-PCR. However, *STI *transcript levels were not significantly increased in *fas1 *and *fas2 *trichomes (Fig. 6). Similar results were obtained for *STI *expression in *fas1 *and *fas2 *seedlings and apices (data not shown). These results suggest that CAF-1 affects STI function instead of modulating *STI *expression.

### H3.2 is up-regulated in *fasciata *mutant trichomes

We previously showed that transcription of the gene for replacement histone variant H3.2 was upregulated in *fas1-1*, *fas2-1 *and *msi1-as *seedlings [40]. H3.2 is incorporated by a CAF-1 independent pathway into nucleosome of chromatin found mostly in transcriptionally active, less compact chromosome regions (reviewed by [4,56]). We asked whether altered trichome differentiation in CAF-1 mutants was correlated with increased expression of *H3.2 *in trichomes. RNA was extracted from trichomes of wild-type, CAF-1 mutants and *msi1-as *plants, and mRNA levels of the H3.2 gene *At1g13370 *were determined by quantitative RT-PCR. This analysis showed that *H3.2 *transcript levels were indeed increased by about 100-fold in trichomes of CAF-1 mutants and *msi1-as *plants (Fig. 6B). These results show that loss of CAF-1 function causes increased expression of *H3.2 *not only in whole seedlings but also in trichomes. Thus, it is likely that chromatin of CAF-1 mutant trichomes contains increased amounts of the H3.2 variant histone.

## Discussion

Trichome cell specification and maturation provide a good model system to study cell differentiation in *Arabidopsis*. Analysis of trichome differentiation has revealed a complex gene network that directs and controls the cell determination, specification and differentiation process [48,57,58]. Here, we report the effects of mutations in the chromatin remodeling complex CAF-1 on trichome development and the genetic interaction of CAF-1 mutant alleles with the trichome regulators *GL3*, *STI *and *KAK*.

**Figure 7:**
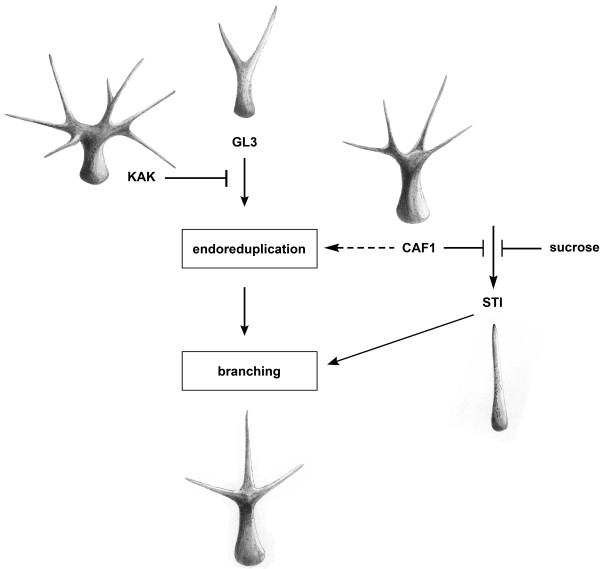
**Model of CAF-1 function in trichome branching**. The initiation of branching is regulated by two independent pathways. In the first pathway, Gl3 is a postive regulator and KAK is a negative regulator. In this pathway, endoreduplication triggers trichome branching. In the second pathway, STI is a positive regulator and CAF-1 is a negative regulator. Exogenous sucrose can partly substitute the negative function of CAF-1. CAF-1 is also required for extensive endoreduplication such as in the *kak-2 *mutant. Images represent trichome phenotypes in the respective mutants.

Because CAF-1 mutants have increased trichome branching but normal endoreduplication [44], CAF-1 limits branching during trichome maturation independent of endoreduplication. Genetic evidence suggests that CAF-1 acts parallel to the *GL3-KAK *pathway (Fig. 7), which promotes trichome branching through the control of endoreduplication ([44,59], this work). Nevertheless, CAF-1 is needed for the *GL3-KAK *pathway to function normally, because the *kak *phenotype is partially suppressed in *kak-2 fas2-1 *double mutants. The *kak-2 fas2-1 *double mutants do not only have less trichome branching but also a lower DNA content than *kak-2 *single mutants. These results suggest that CAF-1 is needed for the increased endoreduplication cycles in *kak-2 *trichomes. One possible explanation for this observation is that the slower progression through the S phase in the mitotic cell cycle, which we proposed for CAF-1 mutants earlier [40], impedes the increased endoreduplication activity in *kak-2 *mutant trichomes. In seedlings and leaves, CAF-1 restricts endoreduplication [34,42-44], and it is possible that lack of CAF-1 triggers additional endocycles in certain cell types with low endoreduplication, but that CAF-1 is also needed to sustain multiple rounds of endocycles in cells types with high endoreduplication such as *kak-2 *trichomes.

Exogenous sucrose alleviates the CAF-1 mutant trichome branching phenotype and weakly suppresses trichome branching in wild-type plants. Since the branching phenotype of CAF-1 mutants grown on soil, which constitutes a less defined but rich medium, was much more similar to the trichome phenotype of CAF-1 mutants grown on sorbitol than on sucrose (data not shown), we suggest that the suppression of trichome branching results from sucrose signaling rather than a starvation effect. Sucrose is known as a potent signaling molecule that controls gene expression, cell cycle and development [50,51]. However, to our knowledge no effect of sucrose on trichome development has been reported before. Sucrose promotes cell cycle progression [60] and can induce endoreduplication [61], but these effects most likely do not explain the observed reduced trichome branching. More rapid progression through the cell cycle and faster growth on sucrose-containing medium could amplify defects associated with chromatin assembly during S-phase in CAF-1 mutants. We found that sucrose greatly enhances the organ development phenotype of *fas2*-1 in L*er*, and mildly enhances this phenotype of other CAF-1 mutant alleles. We propose that specifically during trichome development, sucrose signals can partially substitute for the CAF-1 requirement by a currently unknown mechanism.

## Conclusion

Together, we observed (i) that CAF-1 mutants in a wild-type background have increased trichome branching but no increased endoreduplication, (ii) that CAF-1 mutants and *gl3 *mutants (defective in the endoreduplication-dependent pathway) show an additive interaction, (iii) that CAF-1 mutants and *sti-56 *null mutants (defective in the endoreduplication-independent pathway) show an epistatic interaction, (iv) that CAF-1 mutants enhance the phenotype of the hypomorphic *sti-40 *allele (partially defective in the endoreduplication-independent pathway) and (v) that CAF-1 mutants and *kak *mutants (defective in the endoreduplication-dependent pathway) do not show an epistatic interaction. We conclude that the most parsimonious model to explain all results is that CAF-1 acts together with *STI *in an endoreduplication-independent pathway that is parallel to the endoreduplication-dependent pathway of *GL3 *and *KAK *(Fig. 7). In addition, while CAF-1 is not needed for the normal endoreduplication in WT trichomes, CAF-1 is needed for the extranumerous rounds of endoreduplication that occur in kak mutants. The genetic evidence places CAF-1 in the same pathway with *STI*, an activator of trichome branching that does not affect DNA content [49]. STI shares sequence similarity with the ATP-binding subunit of eubacterial DNA-polymerase III, but the functional relevance of this similarity has not yet been established, and it is not known if STI is a nuclear protein. CAF-1 does not affect trichome branching by modulating *STI *expression, but acts as a negative regulator in the *STI *pathway (Fig. 7). It is not known how CAF-1 can negatively regulate the *STI *pathway. One possibility is that CAF-1 mediated chromatin assembly and compaction [40] are directly needed for normal trichome maturation. Alternatively, it is possible that CAF-1 represses expression of other, limiting components of the *STI *pathway. CAF-1 mutants have increased expression of *H3.2*, which is incorporated into chromatin independently of CAF-1. If chromatin of other genes in the *STI *pathway was enriched in H3.2, the less stable nucleosomes that are formed as a result could facilitate increased transcription, eventually causing increased activity of the *STI *pathway. In summary, we conclude that CAF-1 is required to support the exceptionally high endoreduplication of *kak-2 *trichomes but not for normal endoreduplication of wild-type trichomes. In wild-type trichomes, CAF-1 restricts the activity of the *STI *pathway.

## Methods

### Plant material and growth conditions

Seeds of Columbia (Col), Landsberg *erecta *(L*er*) and Enkheim (En) *Arabidopsis thaliana *wild-type accessions and of *fas1-1 *(accession En) [13,35], *fas2-1 *(accession L*er*) [13,36], *fas1-4 *(accession Col) [44], *fas2-4 *(accession Col) [44] and *gl3-1 *(accession L*er*) [62,63] mutants were obtained from the Nottingham Arabidopsis Stock Centre. Note that in addition to the used *fas1-4 *allele in Col and described first in [44], another *fas1 *allele was described under the same name (*fas1-4*) by Kirik and collaborators in accession C24 [43]. The *msi1-as *line has been described before [44]. The mutants *kak-2 *(accession L*er*) [52], *sti-40 *(accession L*er*) [55] and *sti-56 *(accession L*er*) [49] were kindly provided by M. Hülskamp. Seeds were sown on sterile basal salts Murashige and Skoog (MS) medium (Duchefa, Brussels, Belgium), which was supplemented with 1% sucrose or 1% sorbitol when required. Plants were analyzed on plates or transferred to soil ("Einheitserde", H. Gilgen optima-Werke, Arlesheim, Switzerland) 10 days after germination. Alternatively, seeds were sown directly on soil. Plants were kept in Conviron growth chambers with mixed cold fluorescent and incandescent light (110 to 140 μmol/m2s, 21 ± 2°C) under long day (LD, 16 h light) photoperiods or were alternatively raised in green houses.

### Analysis of trichome branching

To determine the branching pattern, all trichomes on the adaxial side of the first two leaves of an average of six plants were analyzed.

### Ploidy analysis

Ploidy of trichome nuclei was determined as described [44,64]. Briefly, plant tissue was fixed in FAA (50% ethanol, 5% glacial acetic, 10% formaldehyde) and stained for 90 minutes with 130 μg/ml DAPI in McIlvaines buffer (60 mM citric acid, 80 mM sodium phosphate, pH 4.1). Samples were washed twice (15 minutes and 60 minutes) with McIlvaines buffer, and mounted in McIlvaines buffer with 50% glycerol. DAPI fluorescence was recorded with a MagnaFire CCD camera (Optronics, Goleta, CA), or with an Apogee Alta U32 CCD camera (Apogee Instruments, Roseville, CA). Images were quantified using ImageJ. Total fluorescence of at least 30 representative nuclei per experiment was determined and calibrated using guard cell nuclei (n ≥ 30), which are considered to be strictly diploid [64].

### RNA isolation, RT-PCR and Real Time PCR

RNA was extracted from seedlings as previously described [65]. For RT-PCR analysis, 0.4–1 μg total RNA was treated with DNase I. The DNA-free RNA (0.2 – 1.0 μg) was reverse-transcribed using a RevertAid First Strand cDNA Synthesis Kit according to manufacturer's instructions (Fermentas, Nunningen, Switzerland). Trichomes for RNA extraction were harvested into a few microlitres RNA *later *(Ambion, Austin, TX) and then processed like the other samples. Aliquots of the generated cDNA were used as template for PCR with gene specific primers. For qPCR analysis, the Universal ProbeLibrary system (Roche Diagnostics, Rotkreuz, Switzerland) was used on a 7500 Fast Real-Time PCR instrument (Applied Biosystems, Lincoln, CA). Details of the assays used are in Table 2. Analysis of the results was performed according to the method described by Simon [66].

**Table 2:** qPCR assays.

Gene	Forward primer	Reverse primer	Universal Probe Library probe
*STI*, *At2g02480 *(target gene)	agctgagtttgctgggaaaa	ttttcatctgaaacaacaccaac	#9 (Arabidopsis)
*H3.2*, *At1g13370 *(target gene)	aaccgtcgctcttcgtga	ttggaatggaagtttacggttc	#99 (Arabidopsis)
*PP2A*, *At1g13320*, (reference gene^1)^)	ggagagtgacttggttgagca	cattcaccagctgaaagtcg	#82 (Arabidopsis)

^1) ^[67]

Shown are the analyzed genes, sequences of the primers and the identifier of the corresponding Universal ProbeLibrary probes.

## Authors' contributions

VE performed plant analysis, carried out the molecular and genetic studies and drafted the manuscript. LH participated in the study design and helped to write the manuscript. WG helped to write the manuscript.
